# Using Verb Extension to Gauge Children’s Verb Meaning Construals: The Case of Chinese

**DOI:** 10.3389/fpsyg.2020.572198

**Published:** 2021-02-04

**Authors:** Weiyi Ma, Roberta Michnick Golinkoff, Lulu Song, Kathy Hirsh-Pasek

**Affiliations:** ^1^School of Human Environmental Sciences, University of Arkansas, Fayetteville, AR, United States; ^2^School of Education, University of Delaware, Newark, DE, United States; ^3^Department of Psychology, University of Delaware, Newark, DE, United States; ^4^Department of Linguistics and Cognitive Science, University of Delaware, Newark, DE, United States; ^5^Department of Early Childhood Education and Art Education, School of Education, Brooklyn College, City University of New York, New York, NY, United States; ^6^Department of Psychology, Temple University, Philadelphia, PA, United States

**Keywords:** verb extension, age of acquisition, typicality, verb, Chinese

## Abstract

Verb extension is a crucial gauge of the acquisition of verb meaning. In English, studies suggest that young children show conservative extension. An important test of whether an early conservative extension is a general phenomenon or a function of the input language is made possible by Chinese, a language in which verbs are more frequent and acquired earlier. This study tested whether 3-year-old Chinese children extended a group of familiar verbs that specify various ways to *carry* objects. Shown videos that portrayed typical, mid-typical, or atypical carrying actions (as verified by Chinese adults), children were asked to judge whether they were examples of specific Chinese carry verbs. Children’s verb extensions were mostly limited to typical exemplars, suggesting that an early conservative extension may be universal. Furthermore, extension breadth was related to the onset of verb production: verbs acquired earlier elicited more extension judgments than those acquired later.

## Introduction

Verbs label categories of actions and events, thus giving verbs their economy and power (e.g., Hirsh-Pasek and Golinkoff, 2006). Evidence of verb extension can reveal children’s understanding of the breadth and limits of a verb’s meaning. Consider a verb like *carry*. In English, *carry* can be extended to an action regardless of changes in the agent, object (e.g., a man or woman carrying a bag or a baby), or manner (i.e., how an action is carried out; e.g., “carrying” with both arms or on the back). However, in Chinese, there are more than 20 verbs that describe various ways to carry/hold (e.g., Ma et al., 2009). This paper probes how Chinese-speaking children extend carry verbs to a range of appropriate action exemplars. The use of Chinese allows us to test whether the early conservative extension is a general phenomenon or a function of the input language.

While some studies suggest that 1-year-old children can extend their first verbs (Naigles et al., 2009; but see Tomasello and Brandt, 2009), the majority of studies show that young children are conservative in their verb extensions, reluctant to extend both familiar and novel verbs to new instances (e.g., Bates et al., 1979; Gallivan, 1988; Harris et al., 1988; Behrend, 1990; Tomasello, 1992, 2000; Forbes and Poulin-Dubois, 1997; Theakston et al., 2002; Imai et al., 2008; Maguire et al., 2008; Seston et al., 2009; Tomasello and Brandt, 2009). Although recent studies revealed successful learning and extension of novel verbs in 24‐ to 28-month-olds under experimental conditions (e.g., Yuan and Fisher, 2009; Arunachalam and Waxman, 2010, 2011; Scott and Fisher, 2012), successful learning and extension of novel nouns has been documented as occurring a full year earlier (e.g., Ballem and Plunkett, 2005; Yoshida et al., 2009). Furthermore, children were more reluctant to extend verbs to some semantic components (e.g., changes in the manner of the action) than others (e.g., object changes; Behrend, 1990; Forbes and Farrar, 1995). These findings have led to the *conservative verb extension hypothesis*, which states that children are more conservative in their construals of verb meaning and therefore tend to extend verbs more narrowly than adults do (Seston et al., 2009). This hypothesis is supported by previous research in English-speaking children (Bates et al., 1979; Harris et al., 1988; Tomasello, 1992) but this study examines the generalizability of the conservative extension hypothesis in Mandarin-speaking 3-year-olds.

How do children develop appropriate verb extensions? Manipulating the typicality level of exemplars enables us to explore whether children understand the appropriate range of verb extension. If they do, they should accept typical exemplars more readily than less typical ones. Furthermore, if children’s word extension is limited to typical exemplars, it may suggest that their word knowledge views details as more central than do adults. Indeed, research on the acquisition of words from other form classes shows that children initially employ a prototype framework for word meaning (Barrett, 1986, 1995). For example, young children tend to extend familiar words to prototypical exemplars, and later to less typical exemplars (e.g., Meints et al., 1999). Thus, 18-month-olds linked a familiar noun (e.g., bird) to a less typical exemplar (e.g., ostrich), but 12-month-olds did not (Meints et al., 1999; see also Poulin-Dubois and Sissons, 2002). Research on spatial term learning also shows similar prototype effects in both adults and children (Erreich and Valian, 1979; Hayward and Tarr, 1995; Logan and Sadler, 1996; Meints et al., 2002). For example, 15-month-olds associated spatial terms (e.g., under) with typical (e.g., under the *center* of the table) rather than atypical exemplars (e.g., under the *edge* of the table; Meints et al., 2002).

This typicality effect has also been reported in studies on verb understanding with adults and older children. Using adult participants, Ferretti et al. (2001; Experiments 1 and 2) found that verbs presented in isolation primed typical agents (arresting-cop), patients (arresting-criminal), and instruments (stirred-spoon) rather than atypical verbal arguments. Additionally, Meints et al. (2008) showed that the 24-month-olds English-reared toddlers only accepted typical action-patient pairings (e.g., eating-apple) while 3-year-olds and adults also accepted atypical pairings (e.g., eating-houseplants). Relatedly, it was not until 26 months that English-reared toddlers accepted appropriate but atypical manner variations (e.g., kicking a ball with the heel) as instances of familiar verbs (Forbes and Poulin-Dubois, 1997).

This does not mean, however, that the 26-month-olds have an appropriate understanding of all verb extensions, since knowledge of verbs continues to develop well beyond the preschool years (Seston et al., 2009). Furthermore, toddlers are most conservative when the *manner* of the action varied (e.g., Behrend, 1990). The factors influencing children’s verb extension still remain to be elucidated. It is unclear whether conservative extension – especially along the manner dimension – is a general phenomenon of verb learning or a function of the input language. A study of Chinese children’s verb extension may help to address this question.

Chinese differs from English in significant ways that might impact verb acquisition. For example, verbs tend to occupy the salient utterance-final position more frequently in Chinese infant-directed speech (Tardif et al., 1997), and the utterance-final position can facilitate infants’ speech segmentation (Seidl and Johnson, 2006) – a prerequisite for word learning. Furthermore, as a “pro-drop language,” Chinese allows “argument dropping” – a language use phenomenon clearly observable in Chinese infant-directed speech, as well (Tardif et al., 1997; Ma et al., 2019). Thus, the subject, object, or both can be omitted from a sentence and inferred from the context, thereby increasing the frequency and salience of verbs in speech (Tardif et al., 1997). Higher frequency is related to better word comprehension in children (e.g., Rice et al., 1994; Naigles et al., 2009). Additionally, Chinese tends to be pragmatically biased towards verb usage in infant-directed speech (Tardif et al., 1997). For example, in questioning and answering, while English allows nouns as answers, Chinese requires verbs. Thus, to answer the question “Have you eaten your lunch?” one says in Chinese, “Have eaten.” Furthermore, some of the verbs acquired by children-speaking children early in life refer to highly specific meanings. For example, Chinese has more than 20 verbs for “carry/hold,” each labeling a specific way of carrying/holding (Ma et al., 2009). For example, *bēi* means “to carry on the back,” *bào* means “to carry in one’s arms in front of the body,” duān means “to carry flat on two hands in front of the body.^1^” Highly specific verb meanings may facilitate the process of abstracting the commonalities among action exemplars, thus narrowing the semantic scope of a verb. All these factors may enhance early verb acquisition in Chinese children.

Although the majority of research on children’s learning of *novel* verb showed comparable performance between English‐ and Mandarin-speaking children ranging from 24 months to 5 years of age (Imai et al., 2008; Leddon et al., 2011; read Chan et al., 2011 for counterarguments), the verb-friendliness of Mandarin could facilitate Mandarin-speaking children’s learning and extension of *familiar* verbs. Indeed, using the Chinese MacArthur-Bates Communicative Development Inventory (Chinese CDI; Tardif et al., 2008), a parental checklist of young children’s vocabulary, researchers find that Chinese children produce an average of 49 verbs by 19 months. In comparison, English-reared children do not produce this many verbs until 24 months (Fenson et al., 1994). A verb advantage has also been observed in Chinese-speaking children’s spontaneous speech (Tardif et al., 1999).

Do Chinese children know the appropriate semantic scope of familiar verbs as indexed by their understanding of verb extensions? Little research has examined this question. In English, an earlier age of acquisition (AoA) of a word is related to better word processing in a variety of tasks (e.g., Morrison and Ellis, 1995, 2000; Barry et al., 1997, 2001), including visual and auditory lexical decisions (e.g., Turner et al., 1998; Gerhand and Barry, 1999; Morrison and Ellis, 2000). Therefore, an earlier AoA of Chinese verbs presumably offers children more opportunities to assess the range of appropriate verb extensions.

Chinese carry/hold verbs provide multiple advantages for assessing children’s verb extension, not the least among them is that carrying and holding are frequent and familiar events in children’s lives and among the earliest words in Chinese children’s receptive and productive vocabularies (Hao et al., 2008; Tardif et al., 2008). Furthermore, from a methodological standpoint, carry and hold actions are perceptually visible and can be shown dynamically – an important consideration when testing young children. Additionally, compared with verbs with broader meanings, children may be more likely to have a complete understanding of verbs with highly specific meanings and clearly defined semantic boundaries (Maguire et al., 2008; Seston et al., 2009). Thus, this study tests the conservative verb extension hypothesis by investigating whether Chinese children appropriately extend familiar, highly specific, and frequently used carry verbs.

Imai and colleagues examined verb production in Chinese, using carry/hold verbs, for many of the same reasons (Saji et al., 2011). They asked Chinese-speaking 3-, 5-, and 7-year-olds and adults to describe a range of events by using 13 Chinese carry/hold verbs. Results showed that learning the meaning of a verb induced reorganization of the meaning of related verbs. More surprisingly, the 3-year-olds’ verb uses only overlapped with adults’ 17% of the time, suggesting that young children’s verb knowledge significantly differed from adults’. Furthermore, children even seemed to rely on different elements of the verbs’ meaning than adults. For example, the 3-year-olds gave more weight to the salience and the kind of objects used with the carry/hold verbs than adults did, while placing less weight on the manner in selecting the most appropriate verb for a carrying/holding event.

Saji et al. (2011), however, might have underestimated children’s verb knowledge and sensitivity. Saji et al. (2011) paired a different object with each verb, perhaps biasing children’s attention towards the object (Kersten and Smith, 2002). Additionally, language production requires rapid retrieval and phonological encoding, making the task more difficult for young children than a comprehension or judgment task (e.g., Hirsh-Pasek and Golinkoff, 1996; Golinkoff et al., 2013). When children do not produce a verb, it cannot be concluded that they do not know it. Furthermore, these factors may have the strongest influence on the youngest age group (3-year-olds). Thus, it is still unclear whether 3-year-old Chinese children extend the carry/hold verbs properly.

This study probed 3-year-old Chinese children’s extensions of six Mandarin Chinese carry/hold verbs (*bào*: carry with both arms; *bēi*: carry on the back; *kuà*: carry with the elbow; *līn*: carry with bent fingers; *ná*: carry with hands; *tí*: carry with one arm). All of them refer to specific manners of carrying/holding without requiring certain types of objects. Three-year-olds were tested because they are relatively experienced verb users but their semantic understanding of verbs, especially carry/hold verbs, is still developing, making them an ideal age group to examine the factors that could affect verb meaning acquisition (Seston et al., 2009; Saji et al., 2011). Furthermore, 3-year-olds are familiar with the tested carry/hold verbs based on the Chinese CDI data, thus enabling us to examine children’s extension of familiar verbs. Finally, based on the finding that manner extension is harder than object extension (Behrend, 1990), testing this age group allow us to probe children’s developing sensitivity to manner variation.

Each of the six verbs was portrayed by an actor carrying a bag in three types of events – *typical*, *mid-typical*, and *atypical* – created by changing features of the action (Table 1) and confirmed by asking adults for their judgments. Two puppets each offered a sentence about a carrying event and children were asked to judge which puppet was correct in its use of a particular carry verb. The study addressed whether 3-year-olds’ acceptance of the carrying actions differ among typicality levels. If Chinese children would more readily accept typical than less typical exemplars of familiar verbs, just like their English-speaking counterparts in previous studies (e.g., Meints et al., 1999, 2002, 2008; Poulin-Dubois and Sissons, 2002), this study would support the generalizability of the conservative verb extension.

**Table 1:** Descriptions of the typical, mid-typical, and atypical exemplars of the six carry verbs.

	Typical	Mid-typical	Atypical
*bào*	Carry a bag with both arms close to the body in front of the chest	Carry a bag with both arms close to the body (higher than the chest)	Carry a bag with both arms close to the body (lower than the chest)
*beˉi*	Carry a bag on the back	Carry a bag on (one shoulder across the chest)	Carry a bag on (one shoulder beside the body)
*kuà*	Carry a bag inside the elbow of a bent arm close to the side of the body	Carry a bag on (the forearm of a bent arm)	Carry a bag with (one bent arm with the arm raised as high as the shoulder)
*lĩn*	Carry a bag with bent fingers dangling beside the body	Carry a bag with bent fingers beside the body (not dangling)	Carry a bag in (one hand with the arm down beside the body, not dangling)
*ná*	Carry a bag in one hand with the arm down beside the body	Carry a bag (inside the elbow with one bent arm close to the side of the body)	Carry a bag (on the back)^*^
*tí*	Carry a bag in one hand with the arm down beside the body	Carry a bag in one hand with the arm (as high as the waist)	Carry a bag in one hand and (extend the arm out horizontally)

* *As Chinese adults did not consider this event to be an instance of na2, it was omitted from analyses*.

*The ways in which mid-typical and atypical events distinguish from the typical events are described in parentheses*.

## Materials and Methods

### Participants

Nineteen 3-year-old monolingual Mandarin-speaking children (10 males) at a university preschool (*M* = 40.58 months; range: 39.63–41.50) in China participated. Five additional children also participated but were excluded due to failure to comply with instructions (*n* = 2) or to complete the task (*n* = 3). Children were primarily from middle-class homes with college-educated parents. Before the experiment, parents were asked to indicate whether their children understood and produced the six target verbs. Three of the carry/hold verbs (*bào*, *bēi*, *ná*) were produced by all children, consistent with the Chinese CDI data that more than 50% of the normed sample produced these three verbs before 17 months of age (Tardif et al., 2008). For the other three verbs whose Chinese CDI data were unavailable, mothers reported that 17 children (90%) produced *tí*, 11 children (58%) produced *kuà*, and 10 children (53%) produced *līn*. The rates of children producing each verb suggest that the six verbs fell into two groups: *bào*, *bēi*, *ná*, *tí* were each produced by more than 90% of children, all significantly higher than would be expected by chance based on separate Sign tests (*p*’s < 0.001), whereas *kuà* and *līn* were each produced by 58 and 53% of children, which were not different than chance (*p*’s > 0.64). Therefore, we divided the six verbs into early-acquired verbs (*bào*, *bēi*, *ná*, *tí*) and late-acquired verbs (*kuà*, *līn*) based on the percentage of children who could produce them. The sample size of child participants (*n* = 19) was established by conducting a power analysis using G*Power, based on a medium expected effect size (*f* = 0.25) and a power value of 0.80, and the use of a one-sample repeated measures ANOVA containing one group and six measures (three typicality levels × two AoA sets; Faul et al., 2007). This sample size is also consistent with previous research on children’s comprehension of familiar verbs (e.g., Seston et al., 2009). In addition, thirty Chinese college students (15 males; *M* = 21.9 years; range: 20–24) from a local university provided typicality and linguistic judgments of the stimuli.

### Stimuli

**Figure 1:**
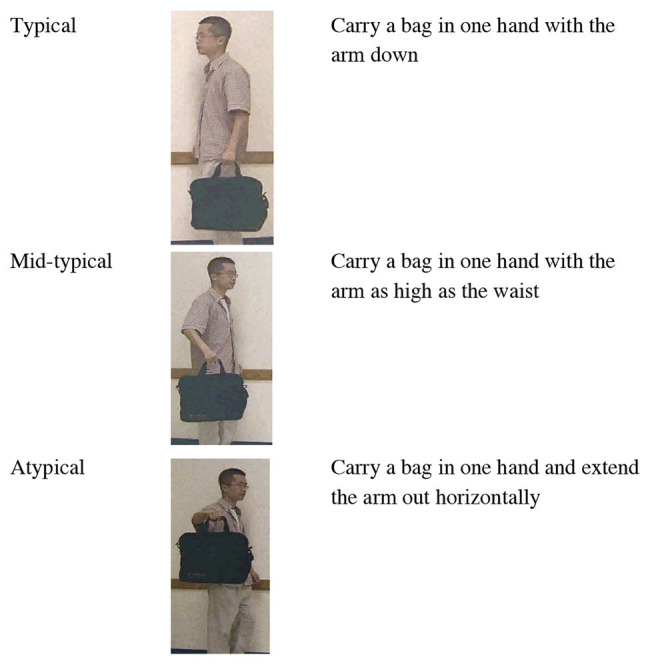
Snapshots of the typical, mid-typical, and atypical action exemplars of *tí* (carry in one’s hand with the arm down). Participants were shown looping videos of dynamic events.

Videos of a human actor carrying a bag were created for six different Mandarin Chinese verbs. For each of the six verbs, three videos varying in the degree of typicality were created; that is, each verb had a typical, mid-typical, and atypical event exemplar. Typicality was manipulated by varying the figure’s manner of motion. This resulted in a total of 18 events, each 8 s in length. The *typical* events depicted the actions according to their dictionary definitions. For example, for the verb *tí*, defined as “carrying with the arm down,” the typical event depicted a human actor carrying a bag with his *arm down*. In the mid-typical event, the actor carried the bag with his *arm up* as high as his waist. In the atypical event, the actor carried the bag with his arm *extending out horizontally* (see Figure 1). Written informed consent was obtained from the individual for the publication of any potentially identifiable images or data included in this article.

### Procedure

#### Adults’ Linguistic and Typicality Judgments

To confirm the appropriateness of the tested carry verbs and the events’ typicality levels, all 18 events were presented to monolingual Mandarin-speaking adults in random orders on a 14-in laptop monitor. Adults were asked to indicate whether an action could be labeled by a particular carry/hold verb. If they answered *yes*, they were asked to rate whether the event was a good exemplar of the particular verb on a 7-point Likert scale (1 = a poor example, 7 = a great example). Adults who indicated that the action could not be labeled by a carry/hold verb were asked to write the verb they thought could label the action.

#### Verb Extension Task

A *trust in testimony method* was used (Koenig et al., 2004). Children were tested individually at their preschool by two native Mandarin speakers. One experimenter (A) manipulated two puppets (a bear and a panda), using a distinct voice for each, to elicit children’s responses: The bear had a low-pitched voice and the panda spoke with a soft, high-pitched voice. Children were told that the puppets needed help with learning some new words. Their responses were recorded by the second experimenter (B) who sat behind the child. All visual stimuli were presented to children on a 14-in laptop computer, while all auditory stimuli were presented in Mandarin child-directed speech by Experimenter A.

##### Familiarization Trials

An experiment started with two familiarization trials, in which *static* images were shown. Experimenter A showed children a picture of a car on the computer and said, “Look, this is a *car*.” Then the bear puppet said, “Yes, it is a *car*,” and the panda puppet followed with, “No, it is not a *car*.” Experimenter A held the two puppets still and asked children, “Which puppet is correct? Can you point to it for me?” If the child did not respond, the experimenter repeated the question once more. The next familiarization trial was conducted in the same way with a picture of an airplane.

The same experimental method was used throughout the entire experimental session. Each puppet was “correct” once and the order of the two trials was counterbalanced. However, children received no feedback on their choices and therefore did not know which puppet the experimenter thought was “correct.” All children succeeded in the two familiarization trials.

##### Training Trials

Next, children participated in four training trials (8 s each) with animated and familiar actions (i.e., flying, swimming, drinking, and sweeping) presented in counterbalanced order. In one training trial, for example, children were shown an animated bird flying. As in the familiarization trials, the experimenter described the action for children using a familiar verb, “Look! The bird is flying.” Each puppet then responded to the experimenter’s description with either, “Yes, the bird is flying,” or “No, the bird is not flying.” The experimenter held the two puppets still and asked the children, “Which puppet is correct? Can you point to it for me?” If children did not respond, the experimenter repeated the question once more. To be included in the final sample, children had to be correct on at least three of the four training trials. Only two children failed to meet this criterion and were excluded.

##### Test Trials

The test trials examined children’s acceptance of the carrying/holding events. The procedure was the same as in the training trials. In each test trial, children were shown a single video clip depicting a typical, mid-typical, or atypical exemplar of a verb. The experimenter described the action for children using a familiar verb (e.g., “Look! He is X-ing a bag.”). Then, each puppet responded to the experimenter’s description with either, “Yes, he is X-ing a bag,” or “No, he is not X-ing a bag.” The experimenter held the two puppets still and asked the children to point to the puppet who was correct. The videos were presented in a pseudorandom order, with the constraints that atypical actions or events labeled by the same verb were never presented consecutively. Across children, test trials were presented in two counterbalanced blocks each containing nine videos of three *carry* verbs: Block A showed *bào, tí, kuà*; Block B showed *ná, bēi, līn*. Children were given a 5-min break between Blocks A and B.

##### Filler Trials

To keep children engaged, a filler trial was inserted after every two test trials and prior to Block B, resulting in a total of eight filler trials involving eight familiar actions: fishing, jumping, climbing, canoeing, playing basketball, kicking a football, licking a lollipop, and pushing a cart. As in training, children were asked to choose the puppet that correctly described the familiar action.

Thus, an experiment consisted of two familiarization trials, four training trials, and two test blocks each containing nine test trials and four filler trials. An experiment lasted about 35 min. Three additional factors were counterbalanced: (1) the hand on which the puppets appeared; (2) the order in which the puppets spoke; and (3) the number of “yes” and “no” responses produced by each puppet.

##### Coding

Each trial received a score of 1 when a child accepted the action as an exemplar of a certain verb or a score of 0 when the child rejected it. Then, at each typicality level, each child had a score of acceptance rate, calculated as the proportion of trials accepted, for the early-acquired (*n* = 4), late-acquired (*n* = 2), and all the verbs (overall), respectively. For example, if a child accepted the typical exemplars of four verbs (e.g., *bào*, *bēi*, *kuà*, and *ná*; *bào*, *bēi*, and *ná* are three of the four early-acquired verbs, whereas *kuà* is one of the two late-acquired verbs), her overall acceptance rate for the typical exemplars would be 0.66 (4/6), and her acceptance rates for the early-acquired and late-acquired verbs were 0.75 (3/4) and 0.50 (1/2), respectively. Only 5% of the trials (19 trials from 11 children) failed to elicit a response, as the children enjoyed the task and were happy to comply. These trials were coded as no-responses in the analyses.

## Results

### Adults’ Linguistic and Typicality Judgments

Preliminary analyses revealed that for all but one action, adults’ acceptance levels were above 90% for the verbs across the typicality levels. That is, in most instances, adults responded to the question of whether an action could be labeled by a particular *carry* verb with a *yes*. The atypical *ná* event was dropped from further analyses as 90% of the adults rejected it as an exemplar of *ná*. Across the six verbs, adults accepted 100% of the typical events, 97% of the mid-typical events, and 95% of the atypical events as exemplifying target *carry* verbs. The differences in acceptance rates among the typicality levels were not significant as assessed by a one-way ANOVA (*F* < 1), suggesting that adults accepted the action exemplars regardless of the typicality levels. However, by comparing adults’ *typicality judgments* as assessed *via* the Likert scale, we found that the action exemplars were rated differently even though they were accepted as examples. Paired-sample *t*-tests with Bonferroni adjustment revealed that adults’ typicality judgments significantly decreased from typical (*M* = 5.98, *SD* = 0.47) to mid-typical (*M* = 4.59, *SD* = 0.42), and from mid-typical to atypical events (*M* = 3.43, *SD* = 0.57; *t*’s > 11.85; *p*’s < 0.001; Cohen’s *d*’s > 2.65). Thus, the typicality levels to which the stimulus events had been assigned were validated by Chinese adults.

### Effects of Typicality and Age of Acquisition on Children’s Verb Extension

To examine how typicality and AoA affected children’s verb extension, a 3 (typicality level) × 2 (AoA: early‐ vs. late-acquired) one-sample repeated-measures ANOVA analyzed children’s acceptance rates of the early‐ and late-acquired verbs. Main effects emerged for typicality [*F*(2,36) = 59.86, *p* < 0.001, *η_p_^2^* = 0.77] and AoA [*F*(1,18) = 4.67, *p* = 0.04, *η_p_^2^* = 0.21]. These two main effects were further examined.

#### The Effect of Typicality

**Figure 2:**
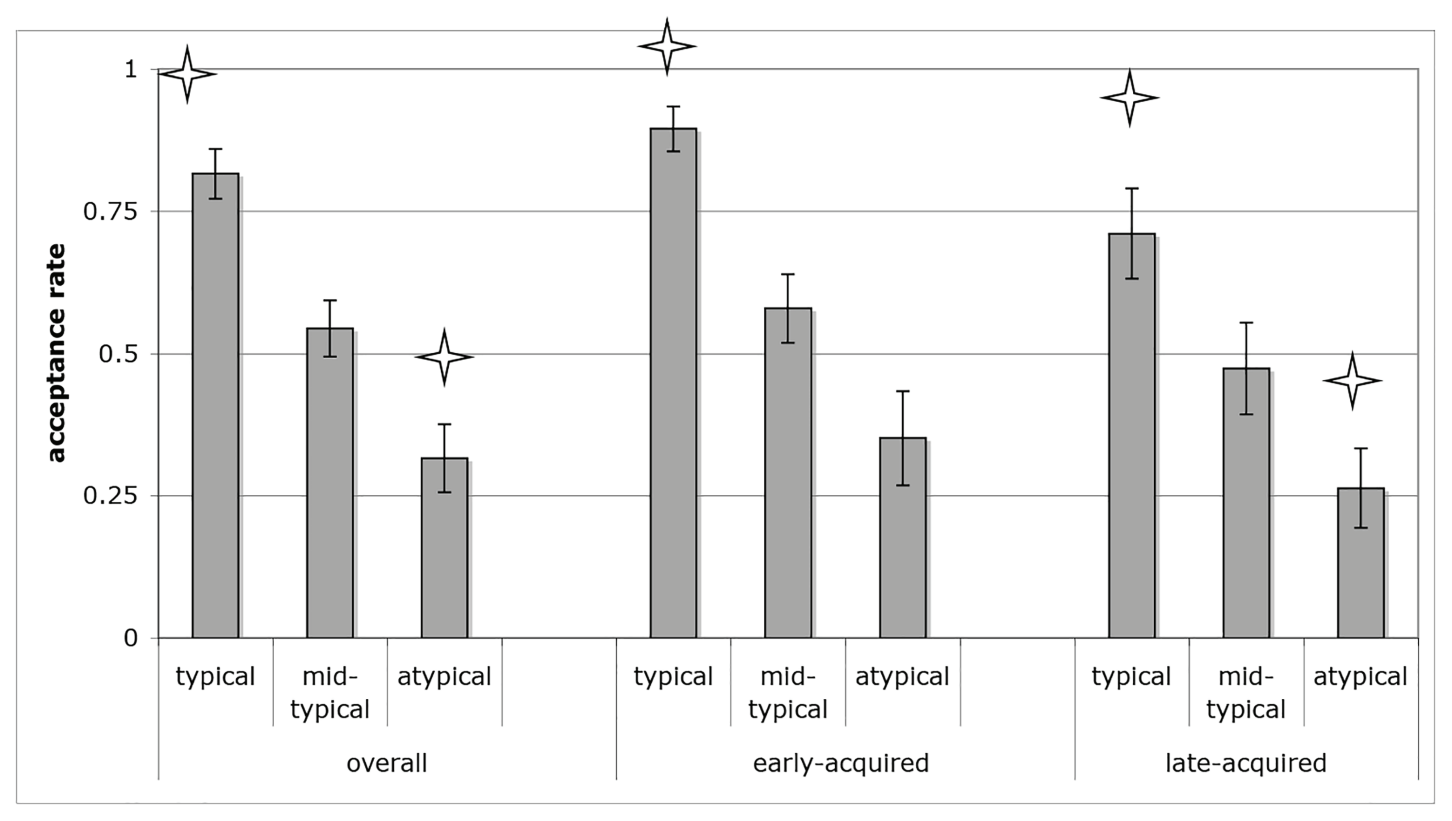
The acceptance rates of the carry events. ^*^p < 0.05 when compared to chance level (0.5).

Paired-sample *t*-tests showed that children’s overall acceptance rates of the typical *carry* events (*M* = 0.83, *SD* = 0.18) were significantly higher than for the mid-typical events [*M* = 0.54, *SD* = 0.21; *t*(18) = 6.90, *p* < 0.001, Cohen’s *d* = 1.58], which were in turn significantly higher than for the atypical events [*M* = 0.32, *SD* = 0.26; *t*(18) = 5.26, *p* < 0.001, Cohen’s *d* = 1.21]. Children’s acceptance rates were also compared to chance (0.50) in separate one-sample *t*-tests. Rates that were significantly higher than chance level would suggest children’s acceptance of the exemplars; rates that were significantly lower than chance would suggest children’s rejection of the exemplars. Results showed that children accepted the typical events [*t*(18) = 8.27, *p* < 0.001, Cohen’s *d* = 3.79], and rejected the atypical events [*t*(18) = −3.08, *p* = 0.006, Cohen’s *d* = 1.41]. Their acceptance of the mid-typical events did not differ from chance [*t*(18) = 0.89, *p* = 0.38]. This pattern held when we examined the early‐ and late-acquired words separately in all the participants. Children accepted typical exemplars of early-acquired [*M* = 0.89, *SD* = 0.17; *t*(18) = 9.94, *p* < 0.001, Cohen’s *d* = 4.56] and late-acquired [*M* = 0.71, *SD* = 0.35; *t*(18) = 2.65, *p* = 0.016, Cohen’s *d* = 1.22] verbs, and rejected atypical exemplars of early-acquired [*M* = 0.35, *SD* = 0.36; *t*(18) = −1.81, *p* = 0.09, Cohen’s *d* = 0.83] and late-acquired [*M* = 0.26, *SD* = 0.31; *t*(18) = −3.38, *p* = 0.003, Cohen’s *d* = 1.55] verbs. Children’s acceptance rates of mid-typical exemplars did not differ from chance for either early-acquired [*M* = 0.58, *SD* = 0.26; *t*(18) = 1.30, *p* = 0.21] and late-acquired [*M* = 0.47, *SD* = 0.35; *t*(18) = −0.33, *p* = 0.75] verbs (Figure 2).

#### The Effect of AoA

The significant main effect of AoA showed that exemplars of early-acquired verbs were accepted at a higher rate than exemplars of late-acquired verbs. The lack of interaction with typicality indicates that this was true at each typicality level (*F* < 1).

### The Relation Between Children’s Production of the Late-Acquired Verbs and Acceptance of Verb Extensions

Given that children differed in whether they produced the two late-acquired verbs, for each late-acquired verb, we divided children into *producers* or *non-producers*. Producers were children whose parents reported that they produced a late acquired verb; non-producers did not yet use it. For *kuà*, all 11 producers and 3 out of 8 non-producers accepted the typical exemplar. A chi-square test revealed that producers of *kuà* were more likely to accept the typical exemplars of *kuà* than the non-producers (*χ*^2^ = 9.33, *df* 1, *p* = 0.002). For *līn*, 9 out of 10 producers and 4 out of 9 non-producers accepted the typical exemplar. A chi-square test revealed that producers of *līn* were more likely to accept the typical exemplars of *līn* than the non-producers (*χ*^2^ = 4.55, *df* 1, *p* = 0.03). For the mid-typical and atypical exemplars, the differences did not reach statistical significance.

## Discussion

This study evaluated whether Mandarin-speaking 3-year-olds extended carry/hold verbs in the way that adults do, or whether children’s extension was more restricted. We presented children with typical, mid-typical, and atypical examples of carrying events accompanied by verbal descriptions and asked them to judge which of two puppets appropriately described these events using carry verbs. Chinese-speaking 3-year-olds, as a group, accepted typical exemplars, were uncertain about mid-typical exemplars, and reliably rejected the atypical exemplars, showing that they were unwilling to extend these familiar verbs to mid-typical or atypical exemplars that vary a figure’s manner of action. Thus, although Chinese children learn verbs earlier and have more verbs in their early vocabularies, they appear to resemble their English-reared counterparts in their similarly conservative verb extensions.

One concern about the task should be mentioned. Children were asked to make metalinguistic judgments – choosing the puppet that correctly described the action – a potentially challenging task. However, children’s ceiling performance in the familiarization, training, and filler trials suggested that they had little difficulty navigating the task. Furthermore, children’s acceptance rates differed systematically across the typicality levels, suggesting that they did not respond randomly. Thus, we believe that this task was valid in gauging children’s verb extensions. What do these results tell us about the acquisition of verb meaning?

In languages such as English and Chinese, there are hundreds of manner verbs (e.g., Talmy, 1985; Slobin, 2003). In English, for example, running, jogging, sprinting, and dashing all name similar actions that vary only in the speed with which they are performed. In Chinese, related but distinctive manner verbs such as the carry/hold verbs studied here are highly productive and appear early in children expressive vocabulary. Children immersed in a manner language may realize that slight differences in a manner can mean that another verb is required to describe the action. This conjecture about manner languages is supported by the research that asked when children begin to construe a novel verb as adults do (Maguire et al., 2010). English-speaking 3-year-olds reliably assumed that a new verb labeled the manner of a novel action, while their Spanish-speaking counterparts more often mapped the new verb to the path of the action. Given a large number of manner verbs in Chinese, Chinese children may be sorting out how much change in a figure’s manner of motion is acceptable before this variation warrants a new verb label. Thus, the under-extension of manner verbs minimizes promiscuous extensions that are likely to be wrong – especially when the class of verbs under consideration has highly specific meanings.

Why do Chinese children, like their English-reared counterparts (e.g., Meints et al., 2008), also limit their verb extensions to mostly typical cases? Typical events may fall more squarely into the category a verb labels than less typical events. Consider the verb *push*, for example. A typical pushing event involves the hands propelling an object forward. A less typical pushing event might involve a bulldozer pushing dirt. Should this exemplar also be described by the verb “pushing”? Children might wait to hear odd events labeled to decide the boundary of the lexical category. Furthermore, typical exemplars might occur more frequently in the world than less typical exemplars. The action of hammering, for example, occurs more often with a hammer than with a shoe, probably causing children to hear a verb applied more frequently to typical than to less typical exemplars. Finally, parents may be biased to label typical rather than less typical exemplars, even when both event types occur, just as they use more basic level (e.g., doggie) than subordinate (e.g., poodle) nouns with young children (e.g., Rosch and Mervis, 1975). The latter two explanations suggest that typicality is closely related to familiarity (Barsalou, 1985). Here all the exemplars were novel, although the typical ones may have been more similar to carry exemplars children had seen before. Thus, the influence of familiarity vs. prototypicality cannot be disentangled in this study.

Children’s reluctance to extend verbs to mid-typical and atypical events suggests that they actually understood the core semantic elements of the tested verbs. However, their verb construals may include more details relative to those of adults’. For example, for the verb *tí*, children only extended to new exemplars when: (1) the bag was carried in the hand; and (2) the arm was straight down. In contrast, Chinese-speaking adults apparently only considered the first element to be the defining feature of *tí*. While evidence suggests that children are aware of the features that may contribute to verb meaning (e.g., Behrend, 1990), perhaps they remain conservative in their verb extensions until they discern which features are criterial. These findings are analogous to how infants initially store the phonological forms of words. Rather than abstracting away the details, infants begin by storing features such as speaker gender and the speaker’s emotional tone along with the acoustic features of a word (Houston and Jusczyk, 2000; Newman, 2008).

Eventually, children do extend verbs as adults do, suggesting that verb meaning may undergo a “characteristic-to-defining shift” just as noun meaning does (Keil and Batterman, 1984). Children begin, for instance, by claiming that the definition of the noun “island” is “a place with palm trees” (i.e., a characteristic), not yet cognizant of the fact that “surrounded on all sides by water” is the only necessary defining feature. The same shift may be seen with verbs: Characteristic features may yield to defining features over time. Multiple exposures to a variety of verb-action combinations may help children come to understand the parameters of verb application (Forbes and Farrar, 1995; Gentner, 2006; Childers and Paik, 2009; Childers, 2011).

Although all children in our sample comprehended the test verbs, not all children produced the late-acquired verbs and this difference was reflected in children’s verb extension. Children who produced a late-acquired verb appeared to be more likely to extend that verb than children who did not yet produce it. One possibility is that the demands of verb production may cause children to notice those event features that influence the use of that verb. However, it is also possible that a better understanding of a verb’s meaning increases the likelihood that it is produced. Future research can determine the direction of causality in this relationship and the changes in the role of typicality over developmental time.

## Conclusion

Despite the putative verb-friendliness of Chinese and the fact that Chinese children have a relative verb advantage compared with their English-speaking counterparts, Chinese-speaking children were reluctant to extend familiar verbs beyond typical exemplars. Thus, it is unlikely that the properties of the input language *per se* are responsible for children’s conservative verb extensions. Children seem to use typical exemplars as their starting point and gradually expand the meaning of verbs to include less typical instances. Learning the meanings of verbs is a prolonged and demanding process (e.g., Bates et al., 1979; Gallivan, 1988; Harris et al., 1988; Tomasello, 1992; Barrett, 1995; Theakston et al., 2002; Seston et al., 2009).

## Data Availability Statement

The raw data supporting the conclusions of this article will be made available by the authors, without undue reservation.

## Ethics Statement

The studies involving human participants were reviewed and approved by Human subject research committee, School of Education, University of Delaware. Written informed consent to participate in this study was provided by the participants’ legal guardian/next of kin. Written informed consent was obtained from the individual for the publication of any potentially identifiable images or data included in this article.

## Author Contributions

WM and RG conceived and designed the study. WM collected data. WM, RG, and LS analyzed the data. All authors contributed to the article and approved the submitted version.

### Conflict of Interest

The authors declare that the research was conducted in the absence of any commercial or financial relationships that could be construed as a potential conflict of interest.

## Footnotes

^1^The strike above a denotes the lexical tone of the Chinese character. There are four basic tones in Mandarin Chinese. Tones are distinctive features in Chinese. For example, *mā* with a high, level tone means mother; *má* with a rising tone means numb; *mă* with a dipping tone means horse; and *mà* with a falling tone means to curse. Furthermore, there are additional instances in which multiple early-acquired Chinese verbs correspond to one verb in English. For example, chuān means ‘to wear something that the body goes through it (e.g., a jacket, pants); daì means “to wear something on the surface of the body (e.g., a hat, a badge, a necklace). In addition, tăng means ‘to lie on the back or side’, and pā means ‘to lie on the stomach’; tī means ‘to kick’, and dēng means ‘to kick with the bottom of the foot.
